# Fluorescence intensity of parathyroid glands in thyroid and parathyroid surgery: a near-infrared autofluorescence study

**DOI:** 10.3389/fsurg.2025.1559274

**Published:** 2025-02-20

**Authors:** Fan Yu, Xiaolei Yi, Zihan Lin, Yinyue Wu, Quanyong Luo, Bo Wu

**Affiliations:** ^1^ Department of Nuclear Medicine, Shanghai Sixth People’s Hospital Affiliated to Shanghai Jiao Tong University School of Medicine, Shanghai, China; ^2^ Department of Ultrasound Medicine, Shanghai Sixth People’s Hospital Affiliated to Shanghai Jiao Tong University School of Medicine, Shanghai, China; ^3^ Department of Thyroid, Breast and Hernia Surgery, Shanghai Sixth People’s Hospital Affiliated to Shanghai Jiao Tong University School of Medicine, Shanghai, China; ^4^ School of Clinical Medicine, The First Clinical College of China Medical University, Shenyang, Liaoning, China

**Keywords:** near-infrared autofluorescence imaging, parathyroid gland, fluorescence intensity, thyroidectomy, primary hyperparathyroidism

## Abstract

**Objective:**

Near-infrared autofluorescence (NIRAF) imaging shows promise in identifying parathyroid gland (PG) during surgery. However, the clinical application of NIRAF faces challenges due to the heterogeneous fluorescence intensity (FI) of PGs observed in different thyroid and parathyroid diseases. This study aimed to evaluate the effectiveness of NIRAF in PG detection and to analyze the FI of PGs in patients with various thyroid and parathyroid diseases.

**Methods:**

A total of 105 patients undergoing thyroidectomy and parathyroidectomy were enrolled. Intraoperative NIRAF imaging was used to detect PGs, and the FI values were quantified using ImageJ software. Normal PGs were grouped according to the pathological results of ipsilateral thyroid diseases. Compare and analyze the FI values of normal and diseased PGs.

**Results:**

A total of 239 PGs were detected during surgery. 225 PGs were identified by NIRAF. The NIRAF identification rate was significantly higher than visual identification (94.1% vs. 81.2%, *p* < 0.001). NIRAF demonstrated high performance in PG identification, with sensitivity, specificity, and positive predictive values and negative predictive values to predict PGs were 95.4%, 77.5%, 90.5% and 88.1%, respectively. The FI of PGs was higher in patients with papillary thyroid carcinoma (1.39 ± 0.21), follicular nodules of thyroid (1.45 ± 0.25), nodular thyroid gland (1.36 ± 0.19) than in those with hyperthyroidism (1.06 ± 0.28) and primary hyperparathyroidism (1.17 ± 0.23). Superior PGs in Stage I exhibited higher FI compared to PGs in Stage II (*p* = 0.025). In Stage II, the FI of inferior PGs was significantly higher than that of superior PGs (*p* < 0.001). The FI of PGs in both Stage I and II was significantly higher than in Stage III.

**Conclusions:**

NIRAF demonstrates high efficiency in identifying PGs across various surgical stages, outperforming conventional visual identification. The FI of superior and inferior PGs exhibits significant variability across different intraoperative stages. Surgeons should exercise caution when identifying PGs in patients with primary hyperparathyroidism and hyperthyroidism, as these conditions are associated with lower FI compared to other thyroid diseases.

## 1 Introduction

In recent years, the incidence of thyroid cancer has been gradually increasing. In 2022, the prevalence of thyroid cancer in China was 33.02 per 100,000, ranking third among all malignant tumors in women (1). Thyroidectomy is the primary treatment for thyroid cancer. The accurate identification and preservation of parathyroid gland (PG) during thyroidectomy are critical to prevent postoperative complications such as hypocalcemia (2, 3). It is widely accepted that the incidence of temporary and permanent hypoparathyroidism after surgery ranges from 5.94% to 67.69% and 0% to 20% respectively (4). However, in some center, the incidence of permanent hypoparathyroidism has been reported to exceed 30% (5). Accurate identification and timely autotransplantation can significantly reduce the incidence of hypoparathyroidism and enhance the quality of life for patients (6).

Near-infrared autofluorescence (NIRAF) imaging has emerged as a promising technique for the intraoperative detection of PGs (7, 8). This non-invasive imaging modality provides real-time visualization of PGs, assisting surgeons in accurately locating and preserving these vital structures during surgery. However, NIRAF also has certain limitations. While NIRAF has demonstrated high sensitivity and precision in identifying PGs in certain patients (9, 10), the fluorescence intensity (FI) can vary significantly due to factors such as surrounding anatomical structures and disease pathology of PG (11, 12). False-positive results may also occur when other tissues, such as thyroid or adipose tissues, exhibit similar autofluorescence characteristics, leading to misidentification of PGs (13). Conversely, false-negative results can arise when PGs do not exhibit sufficient fluorescence, especially in cases of pathological changes like parathyroid adenomas or hyperplasia, which can alter the autofluorescence properties of the tissues (14, 15).

Currently, clinical researches on the variations in FI of normal PGs across different thyroid diseases is relatively limited. In this study, we investigated the FI of PGs in patients with various thyroid and parathyroid diseases, aiming to optimize the application of NIRAF for more effective surgical guidance.

## 2 Methods

### 2.1 Study design

This is a prospective cohort study conducted at the Shanghai Sixth People’s Hospital, involving patients who underwent thyroidectomy and parathyroidectomy between August 2021 and September 2023. All surgeries were performed by the same treatment team with more than 10 years of surgical experience. This study was approved by the human subjects ethics board of Shanghai Sixth People's Hospital [Approval No: 2022-075-(1)]. The written informed consent statements were obtained from all patients in this study.

### 2.2 Inclusion and exclusion criteria

Inclusion criteria were: (i) age >18 years, (ii) condition fulfilled the surgical indications, (iii) first-time thyroidectomy or parathyroidectomy, (iv) intraoperative use of the NIRAF device. Exclusion criteria were: history of thyroidectomy or parathyroidectomy.

### 2.3 Surgical procedure

The commercially available NIRAF device (ARGOS 300PT, Microscopic Intelligence Co., China) was used in the study for detecting PGs. The device consists of a NIRAF camera, a computer system, and a monitor. The NIRAF camera, which emits near-infrared (NIR) light at 785 nm and detects the autofluorescence of PGs at 820 nm, was positioned at a fixed height of 15 cm above the surgical field. The angle of illumination was maintained perpendicular to the tissue surface to ensure consistent light exposure, and the autofluorescence signal was captured as a bright grayscale image displayed on the monitor. Before detecting PGs, ambient light was turned off to minimize interference from external light sources. During the procedure, the NIRAF camera was operated by a single trained assistant, who adjusted its position as needed until the PG was clearly imaged on the monitor to reduce inter-observer variability.

**Figure 1:**
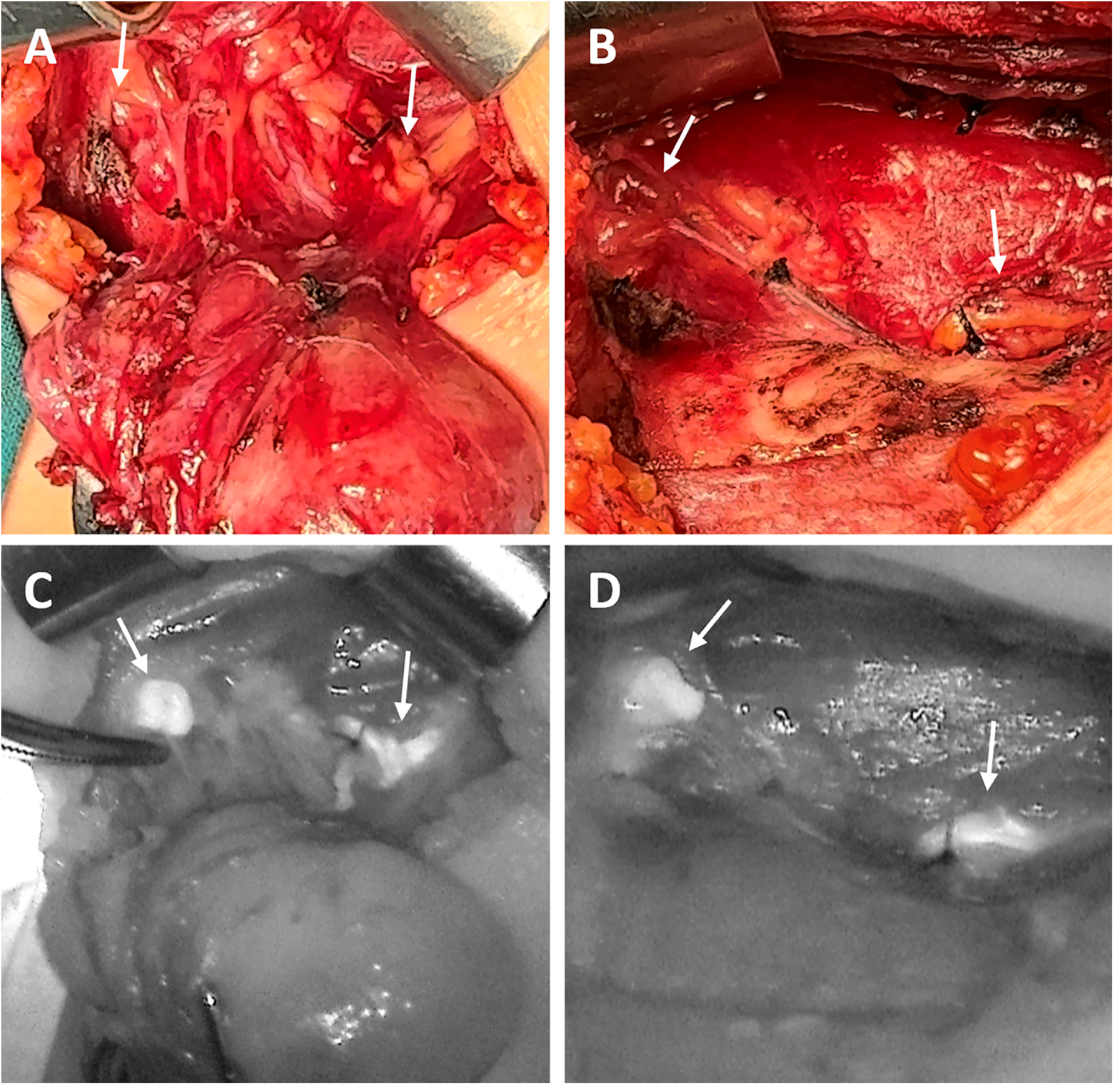
The surgical images **(A,B)** and corresponding NIRAF images **(C,D)** of PG detection in one patient. Two normal PGs (indicated by arrows) with intraoperative autofluorescence with NIRAF in Stage I **(A,C)** and in Stage II **(B,D)**.

**Figure 2:**
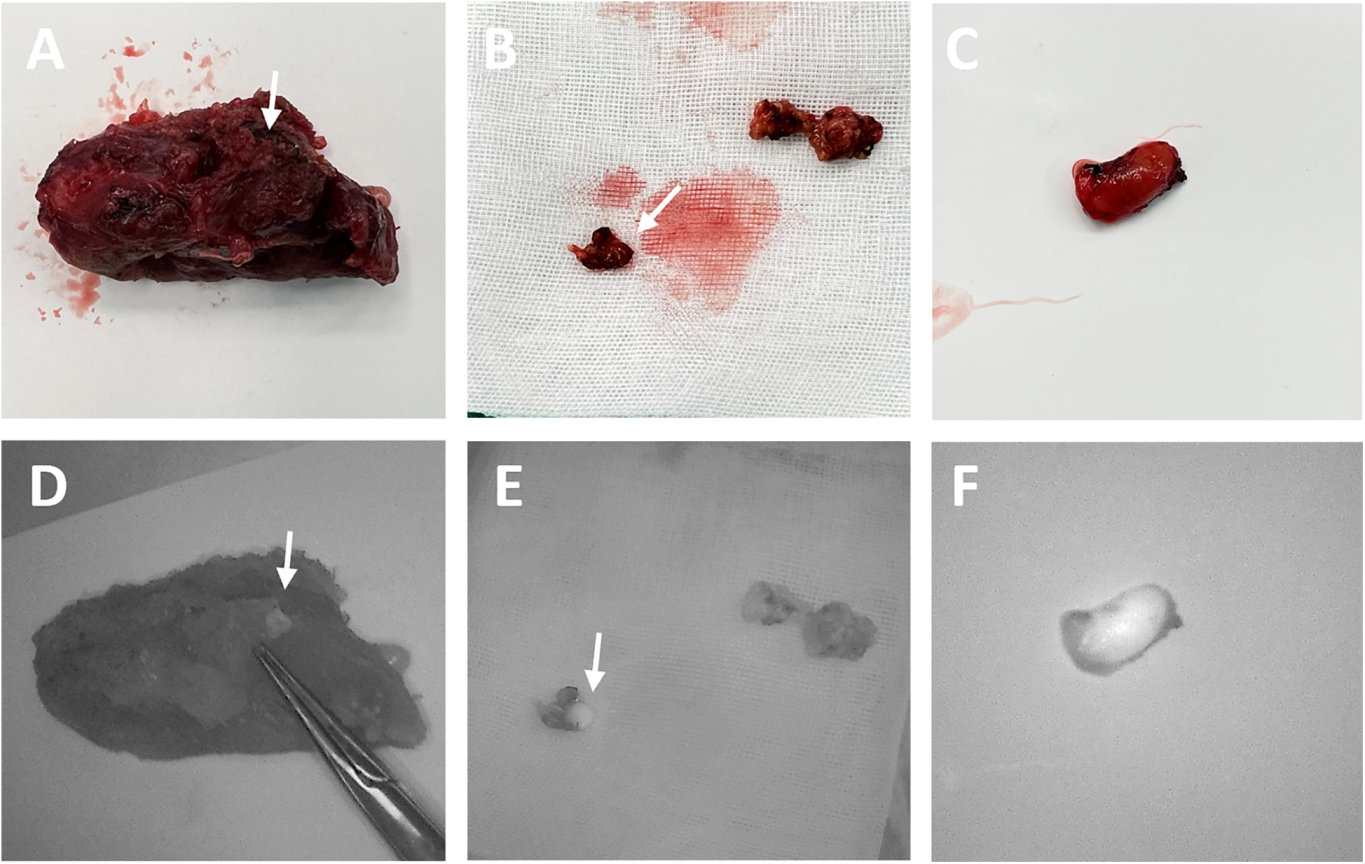
The surgical images **(A–C)** and corresponding NIRAF images **(D–F)** of PG detection in stage III. The inadvertently excised PGs (indicated by arrows) were identified using NIRAF in the isolated thyroid gland **(D)**, central lymph nodes **(E)** and suspected PG tissue **(F)**.

Open thyroidectomy with NIRAF was performed in three stages: Stage I (Surgical field assessment after thyroid membrane dissection), Stage II (Surgical field assessment after thyroid gland removal), and Stage III (Assessment of removed specimens). Stages I and III were used during parathyroidectomy. Only Stage III was used during laparoscopic thyroidectomy. The visualization results from all three stages are shown in Figures 1, 2. If PGs were not detected in one stage, further detection was carried out in the subsequent stage.

PG validation was performed using the immune colloidal gold technique (ICGT), which measures parathyroid hormone (PTH) levels and has demonstrated superior diagnostic accuracy compared to frozen pathological examination (16). ICGT was applied to all specimens suspected to be PGs based on the surgeon's visual inspection or NIRAF detection, including both PGs preserved in the surgical field and those removed during the procedure, to ensure no potential PGs were overlooked. Each excised specimen, including false positive tissues, was pathologically examined according to the 5th edition of the WHO classification of thyroid neoplasms (17). All acquired NIR image data were systematically archived for further analysis.

### 2.4 Image processing

The FI values of the target tissues and background were quantified using ImageJ software. The region of interest (ROI) tool was employed to outline the borders of the PG, thyroid gland, central lymph node, false-positive tissue and background, allowing for the measurement of their mean FI values within the range of 0–255 pixels. The FI values for all target tissues were normalized against the FI of the background (target tissue FI/background FI). The background was defined as the same side of the surgical field in Stage I and II, while a similarly sized region overlying the isolated tissues served as the background in Stage III (10).

### 2.5 NIRAF efficiency

The identification rate for both surgeon's naked eyes and NIRAF were calculated. The number of true positives(TP), true negatives(TN), false positives(FP) and false negatives(FN) identified by NIRAF were recorded in Stage I–III. To evaluate the performance of NIRAF at different surgical stages, the identification results of PGs in each stage were treated as independent samples. Additionally, the sensitivity, specificity, positive predictive value (PPV), and negative predictive value (NPV) were calculated.

The number and location of PGs at different stages were recorded, and the false-positive and false-negative results were analyzed. Each normal PG was grouped according to the pathological findings of the ipsilateral thyroid lobe (the side from which the parathyroid glands were examined) to analyze the FI values.

### 2.6 Statistical analysis

Statistical analysis was performed using SPSS software (version 26.0). Data normality was assessed using the Kolmogorov–Smirnov test. Continuous variables were presented as mean ± standard deviation (SD), while categorical variables were presented as frequencies and percentages. Categorical variables were compared using the *χ*^2^ test or Fisher's exact test, whereas continuous variables were analyzed using the independent *t*-test or one-way ANOVA with the posthoc analysis correction using the Bonferroni test for multiple comparisons.

## 3 Results

The clinical characteristics of the 105 patients are presented in Table 1. A total of 239 PGs were identified during surgery, with 81.2% (194/239) identified visually by surgeons and 94.1% (225/239) detected using NIRAF. Postoperative pathological examination confirmed PGs in 8 patients, with 4 detected in thyroid glands and 4 in central lymph nodes. The NIRAF identification rate was significantly higher than visual identification (*p* < 0.001). Among the detected PGs, 167 were identified in Stage I, 172 in Stage II, and 52 in Stage III. The mis-cut rate of PGs was 7.7% (17/221) and the autotransplantation rate was 16.3% (36/221) in thyroidectomy. A total of 411 true positives, 43 false positives, 148 true negatives, and 20 false negatives were recorded across all stages. Based on these values, the sensitivity, specificity, PPV, and NPV of NIRAF were calculated as 95.4%, 77.5%, 90.5%, and 88.1%, respectively.

**Table 1:** Clinical characteristics of 105 patients in study.

Characteristics	*N* (%)
Sex, *n* (%)	
Male	29 (27.6%)
Female	76 (72.4%)
Age (years, Mean ± SD)	47.48 ± 13.95
Days of hospitalization (days, Mean ± SD)	6.01 ± 2.39
Operation time (minutes, Mean ± SD)	75.48 ± 25.99
Scope of surgery, *n* (%)	
Unilateral	69 (64.1%)
Bilateral	36 (35.9%)
Procedure, *n* (%)	
Thyroid lobectomy	8 (7.6%)
Thyroid lobectomy with CND^a^	52 (49.5%)
Total thyroidectomy	11 (10.5%)
Total thyroidectomy with CND	22 (21.0%)
Laparoscopic unilateral thyroidectomy	2 (1.9%)
Parathyroidectomy	8 (7.6%)
Thyroid lobectomy with CND and parathyroidectomy	1 (1.0%)
Total thyroidectomy with parathyroidectomy	1 (1.0%)

^a^ CND, central neck dissection.

**Figure 3:**
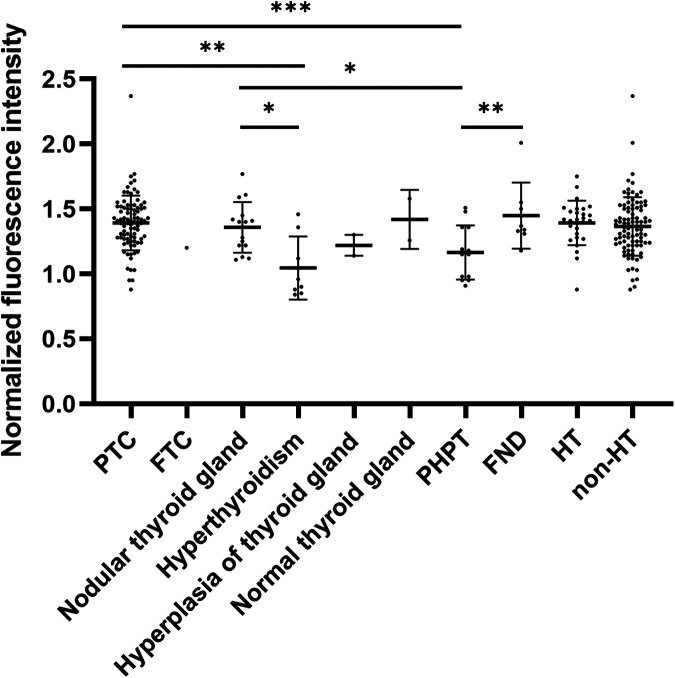
Normalized FI of PGs in ipsilateral thyroid pathology and PHPT. *p < 0.05, **p < 0.01, ***p < 0.001.

According to the thyroid and parathyroid pathological findings from 105 patients, the results included 88 cases of papillary thyroid carcinoma (PTC), 1 case of follicular thyroid carcinoma (FTC), 15 cases of nodular thyroid gland, 8 cases of follicular nodules (FND), 2 cases of normal thyroid, 8 cases of hyperthyroidism, 3 cases of thyroid hyperplasia, and 13 cases of primary hyperparathyroidism (PHPT). The distribution of the normalized FI values for PGs in different diseases is shown in Figure 3 and Table 2. The FI of PGs was significantly higher in PTC and follicular nodules compared to PHPT (*p* < 0.001; *p* = 0.006) and hyperthyroidism (*p* = 0.006; *p* = 0.033). After applying the Bonferroni correction, the comparison between follicular nodules and hyperthyroidism was not statistically significant. Additionally, FI in PGs with nodular thyroid gland was significantly higher compared to hyperthyroidism (*p* = 0.027). No significant difference in FI was observed between patients with Hashimoto's thyroiditis (HT) and those without HT (*p* = 0.902).

**Table 2:** Normalized FI of PGs in different diseases of thyroid lobe and PHPT.

	FI (Mean ± SD)	*N* (%)
Papillary thyroid carcinoma	1.39 ± 0.21	88 (63.8%)
Follicular thyroid carcinoma	1.20 ± 0.00	1 (0.7%)
Nodular thyroid gland	1.36 ± 0.19	15 (10.9%)
Hyperthyroidism	1.06 ± 0.28	8 (5.8%)
Hyperplasia of thyroid gland	1.22 ± 0.08	3 (2.2%)
Normal thyroid gland	1.42 ± 0.23	2 (1.4%)
Follicular nodules of thyroid	1.45 ± 0.25	8 (5.8%)
Primary hyperparathyroidism	1.17 ± 0.23	13 (9.4%)
Hashimoto's thyroiditis	1.41 ± 0.16	28 (23.0%)
Non Hashimoto's thyroiditis	1.41 ± 0.24	94 (77.0%)

In Stage II, inferior PGs exhibited significantly higher FI than superior PGs (*p* < 0.001). The FI of PGs was also significantly higher in Stage I and Stage II compared to Stage III. The FI of PGs was significantly higher than that of the thyroid gland in Stage I (*p* < 0.001). The FI values of PGs, thyroid glands, and central lymph nodes across the three stages are detailed in Table 3.

**Table 3:** Normalized FI of PG, thyroid gland, central lymph node in different stages.

	FI in Stage I (Mean ± SD)	FI in Stage II (Mean ± SD)	FI in Stage III (Mean ± SD)	*p* value (Stage I vs. II)	*p* value (Stage I vs. III)	*p* value (Stage II vs. III)
PGs	1.47 ± 0.27*	1.42 ± 0.26	1.17 ± 0.24^†^	0.057	<0.001	<0.001
Superior PGs	1.45 ± 0.25*	1.37 ± 0.24	1.17 ± 0.23^†^	0.025	<0.001	0.001
Inferior PGs	1.50 ± 0.30*	1.48 ± 0.27^#^	1.17 ± 0.23^†^	0.667	<0.001	<0.001
Thyroid gland	1.07 ± 0.14	—	0.79 ± 0.10	—	<0.001	—
Central lymph node	—	—	0.82 ± 0.10	—	—	—

* *p* < 0.001 compare with thyroid gland group in Stage I.

^#^ *p* = 0.001 compare with superior PGs group in Stage II.

^†^ *p* < 0.001 compare with thyroid gland and central lymph node groups in Stage III.

False positives included tissues from PTC, normal thyroid, nodular thyroid glands, scabs, adipose tissue, and central lymph nodes. The FI of scabs and nodular thyroid tissue was significantly higher than that of other tissues (Table 4). False-negative PGs were observed in 7/88 (7.95%) PTC cases, 2/8 (25.0%) hyperthyroidism cases, 1/8 (12.5%) FND cases, and 2/13 (15.38%) PHPT cases.

**Table 4:** Normalized FI of false-positive tissues.

	FI (Mean ± SD)	*N* (%)
Normal thyroid gland	1.05 ± 0.15	5 (11.6%)
Nodular thyroid gland	1.21 ± 0.31^#^	3 (7.0%)
Central lymph node	1.08 ± 0.10	6 (14.0%)
Adipose tissue	1.06 ± 0.10	14 (32.6%)
Mixture of adipose tissue and central lymph node	1.08 ± 0.10	6 (14.0%)
Scab from energy devices	1.42 ± 0.09*	3 (7.0%)
Papillary thyroid carcinoma	1.04 ± 0.08	6 (14.0%)

^#^ *p* < 0.05 compared to thyroid gland, PTC, adipose tissue and central lymph node groups.

* *p* < 0.001 compared to thyroid gland, PTC and adipose tissue groups.

## 4 Discussion

The identification and preservation of PGs are crucial in thyroid surgery to prevent postoperative complications such as hypoparathyroidism and hypocalcemia. Due to their delicate structure and similarity to surrounding tissues, PGs are particularly vulnerable to injury during surgery (4, 18). While visual identification remains the primary method for detecting PGs, its accuracy highly depends on the surgeon's experience (5), which reported rates ranging from 61% to 93.6% (19–21). Even experienced surgeons may miss PGs during the procedure. Advanced techniques such as nano-carbon technology have improved PG identification (22, 23), but their accuracy is compromised in cases of lymphatic or tumor obstruction (23). Additionally, the high cost and reliance on visual identification limit their widespread use. Frozen section pathological examination and ICGT have demonstrated high accuracy and are considered reliable adjuncts in PG identification (16, 24). Therefore, we utilize ICGT to validate suspected PG in our study.

NIRAF imaging has emerged as a promising technique for real-time, non-invasive PG identification. Paras et al. demonstrated that PGs exhibit maximum FI at 820–830 nm under 785 nm NIR light excitation (25). This technology enables surgeons to detect PGs at any time, facilitating *in situ* preservation or timely autotransplantation, thereby reducing complications (26). Indocyanine green (ICG) is the natural dye, which can enhance the FI of PGs when combined with NIRAF (27). However, the low tissue specificity of ICG may cause fluorescence signals in thyroid and surrounding tissues, thereby interfering with the accurate identification of PGs by NIRAF (28). In our study, we did not employ the combination of ICG and NIRAF, focusing instead on the intrinsic fluorescence properties of NIRAF for PG identification.

Our study confirmed that NIRAF significantly improves PG identification rates compared to visual inspection, with a sensitivity of 95.4% and a positive predictive value of 90.5%, consistent with previous studies (19, 29, 30). In this study, the FI of PGs was based on the pathological results of the ipsilateral thyroid lobe (the side from which PGs were examined) and PHPT themselves rather than patients' number. This approach ensures consistency in the analysis and minimizes potential confounding effects from the contralateral thyroid lobe, enabling a systematic investigation into the relationship between the pathology of thyroid and parathyroid diseases and PG autofluorescence. However, false-negative results of PGs were observed, particularly in cases with PTC (7/88, 7.95%), hyperthyroidism (2/8, 25.0%), FND (1/8, 12.5%), and PHPT (2/13, 15.38%). To further investigate, we calculated the normalized FI values of PGs across different thyroid diseases. The blood supply to PGs is primarily provided by the inferior thyroid artery or directly from the thyroid gland itself (31). Therefore, PGs associated with hyperthyroidism, thyroid hyperplasia exhibited lower FI, likely due to hypervasular nature of these tissues. Previous study have demonstrated that blood has a higher absorption coefficient for NIR light than other tissues (11), increased vascularity in these conditions may enhance light absorption, reducing FI of PGs. These findings emphasize the need for cautious interpretation of NIRAF images in hyperthyroidism and thyroid hyperplasia, as lower FI may increase the risk of inadvertent PG excision.

Additionally, NIRAF occasionally identified false-positive tissues, such as thyroid gland, lymph nodes, adipose tissue, and scabs from energy devices. We calculated the FI values of different false-positive tissues separately and found that the scabs from energy devices with the highest FI value. Due to the discrepancy between the morphology and location of scabs and PGs, they are easily distinguishable by surgeons. However, in some patients, false positives may occur where PTC or nodular thyroid gland is adjacent to or protrudes from the thyroid peritoneum. Additionally, false positives can arise from adipose tissues and lymph nodes, introducing challenges in visual identification. Therefore, when autofluorescence is detected on the thyroid, it may be advisable to incise the thyroid surface to determine whether the fluorescence originates from the thyroid tumor or PGs within the gland (32, 33). Furthermore, in cases where the presence of false positive tissues remains uncertain, ICGT technology provides a reliable approach to reduce the risk of misjudgment.

We also observed dynamic FI variations across surgical stages. Superior PGs showed higher FI in Stage I, while inferior PGs exhibited increased FI in Stage II. Currently, there is limited research addressing the FI differences between superior and inferior PGs. We hypothesize that, given the FI of PGs remains consistent post-excision, the *in vivo* variations may be linked to factors such as adipose tissue exposure or vascularization in the surgical field (11). Superior PGs, being more fixed and less covered, are readily identifiable by NIRAF early in surgery. In contrast, inferior PGs, often obscured by adipose tissue and lymph nodes, exhibit lower FI initially but show increased FI as surgery progresses and these tissues are dissected. This dynamic change likely reflects improved visualization and vascular exposure of inferior PGs, highlighting the importance of meticulous dissection and real-time FI monitoring for PG identification and preservation. We observed the lower FI in Stage III compared to Stages I and II, which we attribute to changes in the reference background. During Stage III, the PGs are typically situated within surrounding tissues, such as lymph nodes, thyroid glands, or adipose tissues. These tissues, which dominate the field of view, exhibit inherent autofluorescence that interferes with the FI of the PGs, complicating their detection. Therefore, careful interpretation of NIRAF signals is essential during Stage III to minimize the risk of missing PGs.

Interestingly, both HT and non-HT patients exhibited higher FI of PGs compared to other diagnoses, although no significant difference was observed between HT and non-HT groups. Given that HT was not the primary surgical indication and both groups encompassed heterogeneous diagnoses of thyroid diseases, we hypothesize that the higher FI may be due to confounding factors. Our preliminary analysis suggests that HT is not the significant determinant of PG autofluorescence. Future studies with larger sample sizes and a specific focus on thyroiditis are warranted to elucidate the relationship between HT and PG autofluorescence.

To our knowledge, this is the first study to classify PG FI values based on the pathological results of the ipsilateral thyroid lobe, providing a comprehensive understanding of autofluorescence variations across different thyroid diseases. However, our study has limitations, including a small sample size, particularly for 1 case of FTC and 2 cases of normal thyroid glands. These cases were excluded from the final analysis, as their inclusion would not provide meaningful statistical power or reliable results. Secondly, this was a single-center study, which may introduce center-specific biases. Future research should aim to include a more diverse range of participants from multiple centers to validate and extend the current results. Additionally, further investigation into the molecular mechanisms underlying the variations in fluorescence intensity could provide valuable insights into the autofluorescence properties of parathyroid tissues.

## 5 Conclusions

NIRAF has demonstrated high efficiency in identifying PGs at various stages in surgery, surpassing conventional visual identification by surgeons. The FI of superior and inferior PGs exhibits significant variability across different intraoperative stages. Special attention during surgery is required when identifying PGs in patients with PHPT and hyperthyroidism, as the PGs exhibit lower FI compared to those in patients with PTC or nodular thyroid disease.

## Data Availability

The raw data supporting the conclusions of this article will be made available by the authors, without undue reservation.
